# Belladonna in Dysmenorrhœa and Neuralgia

**Published:** 1844-07

**Authors:** 


*Belladonna in Dysmenorrhœa and Neuralgia.*—Dr. Golding Bird, at a late meeting of the London Medical Society, said he would make some practical observations on a subject which had been mentioned sometime since, the treatment of dysmenorrhœa, and for which the president had recommended veratria frictions over the loins. It is a very frequent complaint among unmarried women, so much so that Dr. Bird believed that every third case among them in practice was one of this malady. He was of opinion that an irritable uterus was connected with the various pains and aches which young people suffer from. The plan of treatment which he was about to recommend, was certainly empirical, but it was also very successful; he received it from his brother, when he was attending the practice of Dr. Roe in Westminster hospital. In treating these cases, he drew two distinctions founded on the seat of pain. In the first, there is experienced more agonizing pain at the lower part of the abdomen, just over the situation of the uterus: in the second class, the pain is composed of both. The first of these is the most intense, and the most frequently met with. He (Dr. Bird) has seen a lady roll off the sofa on to the ground in her agony of suffering. In cases where the pain is seated over the region of the uterus, in the lower part of the abdomen, he has given the belladonna internally, taking care that the extract is properly prepared, and has invariably found it successful; great relief is experienced at the next menstrual period; greater still at the succeeding one, and a cure is finally accomplished. The mode of exhibition must be modified according to the temperament of the patient. In young women, of a pale leucophlegmatic habit, with a pallid or chlorotic face, it should be combined with the sulphate of zinc, in the proportion of five grains of extract of belladonna to twenty grains of the sulphate of zinc made into twenty pills, one to be given every two or three hours, immediately on the accession of the pain at a menstrual period, until the pain ceases. For girls of a plethoric habit, with a red face, the extract should be combined with ipecacuanha, in the proportion of five grains of extract to ten grains of ipecacuanha, made into twenty pills, administered in the same manner as the preceding. The previous treatment should consist in clearing out the bowels, improving the general health, and removing cachexia, and an aperient of castor oil or olive oil, should be given before the occurrence of the menstrual period. This treatment is to be repeated at each menstrual period, and will always effect a cure if there be not any organic disease, and the dysmenorrhœa be unattended with the discharge of shreds of false membrane. By removing the irritable condition of the uterus, and curing the dysmenorrhœa, the pseudo-pleurisies and pseudo-peritonitis, will soon get well. The cases in which the pain is seated, chiefly in the loins, do not yield to the employment of belladonna. This Dr. Bird considered was owing to congestion of the uterus. Fortunately the cases that are remediable are the most frequent.

Mr. Pilcher mentioned that he had tried belladonna in certain cases of neuralgia of the face, but not with the satisfactory result he had been led to expect. This he thought might have been owing to the bad preparation of the drug. He had since turned his attention to the exhibition of quinine, with which he had successfully treated two cases of neuralgia of the face and breast. Some years ago he gave a nephew of his, a boy six years of age, then laboring under a severe attack of pertussis, a combination of the extract of belladonna, with ipecacuanha, in the proportion of a grain of the extract to half a grain of ipecacuanha, three or four times a day, with marked benefit, the paroxysms being materially shortened, and great relief afforded. No narcotism was produced. In another case, that of a delicate little girl, it also afforded relief, but it produced dangerous symptoms of narcotism, and was consequently given up. He had directed it in several cases of tic douloureux, but it had not produced the effect he had anticipated. In the case of a barber's clerk, whose health was broken by neuralgia, caused by a decayed tooth, which had long since been extracted, he had prescribed a combination of three grains quinine and a quarter of grain of morphia every four hours, with very great relief. He had seen the man only twice, and he had not had any pain for forty-eight hours. In the case of a woman, also suffering from a similar affection, the same combination and smaller doses had afforded great relief. In neuralgia of the breast, where quinine fails, he had found benefit from Batty's liquor cinchonæ.

Dr. Waller observed that Dr. Bird's recommendation of belladonna in these cases should not be lost sight of by him. He had been in the habit of prescribing it in such cases by itself with advantage, but could not effect a permanent cure. He had also employed the extract externally, spread on leather, with a rim or margin of adhesive plaster to make it adhere. In painful dyspepsia, gastrodynia, he had been in the habit of prescribing it with benefit. In one very obstinate case he had combined it with large doses of Scheele's prussic acid. He had also ordered it in cases of anamalous pains, of which he could not well tell the origin, and in one case in particular he had made a lucky hit, for he could not call it anything else. An old lady, who had a curious pain at the back of the shoulder, from which she had been suffering for several months, was relieved by it in forty-eight hours. He thought the distinction drawn by Dr. Bird with respect to the seat of pain in dysmenorrhœa, a very important one; in cases where the pain is felt principally in the loins, he had found more benefit from the application of leeches inside the vulva than from anything else.—*London Med. Times.*

